# Chromatin Regulator-Related Gene Signature for Predicting Prognosis and Immunotherapy Efficacy in Breast Cancer

**DOI:** 10.1155/2023/2736932

**Published:** 2023-01-30

**Authors:** Dongxu Feng, Wenbing Li, Wei Wu, Ulf Dietrich Kahlert, Pingfa Gao, Gangfeng Hu, Xia Huang, Wenjie Shi, Huichao Li

**Affiliations:** ^1^Department of General Surgery, Chongming Hospital Affiliated to Shanghai University of Medicine and Health Sciences, Chongming District, Shanghai 202150, China; ^2^University Clinic for General, Visceral, Vascular-and Transplantation Surgery, Faculty of Medicine, Otto-von-Guericke-University, Magdeburg 39120, Germany; ^3^University Hospital for Gynaecology, Pius-Hospital, University Medicine Oldenburg, Oldenburg 26121, Germany; ^4^Department of Thyroid Surgery, The Affiliated Hospital of Qingdao University, Qingdao 266555, Shandong, China

## Abstract

**Background:**

Many studies have found that chromatin regulators (CRs) are correlated with tumorigenesis and disease prognosis. Here, we attempted to build a new CR-related gene model to predict breast cancer (BC) survival status.

**Methods:**

First, the CR-related differentially expressed genes (DEGs) were screened in normal and tumor breast tissues, and the potential mechanism of CR-related DEGs was determined by function analysis. Based on the prognostic DEGs, the Cox regression model was applied to build a signature for BC. Then, survival and receiver operating characteristic (ROC) curves were performed to validate the signature's efficacy and identify its independent prognostic value. The CIBERSORT and tumor immune dysfunction and exclusion (TIDE) algorithms were used to assess the immune cells infiltration and immunotherapy efficacy for this signature, respectively. Additionally, a novel nomogram was also built for clinical decisions.

**Results:**

We identified 98 CR-related DEGs in breast tissues and constructed a novel 6 CR-related gene signature (ARID5A, ASCL1, IKZF3, KDM4B, PRDM11, and TFF1) to predict the outcome of BC patients. The prognostic value of this CR-related gene signature was validated with outstanding predictive performance. The TIDE analysis revealed that the high-risk group patients had a better response to immune checkpoint blockade (ICB) therapy.

**Conclusion:**

A new CR-related gene signature was built, and this signature could provide the independent predictive capability of prognosis and immunotherapy efficacy for BC patients.

## 1. Introduction

Breast cancer (BC) is a common cancer in the world [1]. Although the widespread use of adjuvant chemotherapy and hormonal drugs has reduced mortality in BC patients, there are still individual differences in treatment outcomes and different clinical benefits for BC patients [2]. Fortunately, with the continuous updating of new therapies, the use of cancer biomarkers has become an aid in BC diagnosis, prognosis, treatment response prediction, and disease monitoring during and after treatment [3]. Nowadays, many researchers have tried to use various bioinformatics techniques to identify the biomarkers or build the risk model in BC and achieved good research results, such as the machine learning model in predicting the immune subtype [4] and the eight-lncRNA prognostic model [5]. Therefore, in order to provide a more effective prediction of survival in tumor patients, continuous efforts are needed to identify new prognostic key molecules and explore their prognostic values.

Chromatin regulators (CRs) are essential upstream regulatory factors in epigenetics that can cooperate to connect the organizational scales of chromatin from nucleosome assembly to the establishment of functional chromatin domains [6]. CRs can highly regulate chromatin structure by four broad classes of nuclear factors, including histone variants, histone chaperones, chromatin remodelers, and histone modifiers [7]. In human cancer pathogenesis, it has been found that the function of CRs is frequently disrupted by genetic mutations and epigenetic alterations, resulting in perturbed gene expression profiles. The role of CRs in cancer is complex and highly specific [8]. To begin with, the carcinogenic effects of chromatin regulators are well established. Meanwhile, recent new studies have demonstrated their tumor suppressive properties in the regulation of multiple cellular processes [9]. For example, MLL3/4, one of the chromatin regulators, may exert broad tumor suppressor effects in various cancers [10]. In addition, growing evidence has shown that CRs and tumor prognosis are closely related. In cervical cancer, 57 overexpressed chromatin regulators were identified to have prognostic significance [11]. Similarly, CR-related genes were found to be significantly associated with postoperative outcomes in astrocytomas [12]. However, CRs affecting prognosis of breast cancer is still lacking more understanding and research.

The tumor microenvironment (TME), consisting of extracellular matrix, stromal cells, and immune cells [13], is important in cancer initiation, progression, and therapy [14]. With the emergence of new technologies, immunotherapy has become a widely concerned research direction for cancer treatment, which aims to use the immune system as a tool for treating oncological diseases [15]. The effect of immunotherapy is associated with the TME, especially the tumor-infiltrating immune cells (TICs) [16]. Currently, there are 3 types of immunotherapies that target tumor-specific T cells, including immune checkpoint blockade (ICB), adoptive cellular therapies, and cancer vaccines [17]. In immune checkpoint blockade therapy, multiple cancers have been treated with two different antiprogrammed cell death protein 1 (PD1) drugs (nivolumab and pembrolizumab) [18] and anticytotoxic T lymphocyte-associated antigen 4 (CTLA4) (ipilimumab) [19]. The use of therapeutic antibodies does provide clinical benefit to a small patient population, but adverse effects associated with immune checkpoint blockade complicate immunotherapy and limits its use in cancer patients [20]. Therefore, in order to promote the efficacy of ICB therapy, it is necessary to identify and explore the predictive biomarkers for immune checkpoint-blocking therapies [21, 22].

In this work, we investigated the potentiality of CR-related DEGs as prognostic markers in BC through several bioinformatic analyses. Furthermore, CR-related DEGs were successfully employed to construct a six-gene prognostic model and a new CR-related gene signature for predicting patients' outcome and immunotherapy efficacy.

## 2. Materials and Methods

### 2.1. Data Collection

The training set was downloaded from the The Cancer Genome Atlas (TCGA) database (*n* = 1167; 113 normal samples vs. 1054 tumor samples). The validate set was obtained from GSE20685 (*n* = 327) [23]. Considering that a patient's follow-up for less than 30 days is too short to evaluate the prognosis, we excluded those patients for a more accurate evaluation. The complete clinical information of patients is shown in Supplementary Table 1. Lastly, there were 870 chromatin regulator (CR)-related genes obtained from previous research [6].

### 2.2. Identification of CR-Related DEGs

First, the data of the training set were normalized with the log2 transformation. Next, the expression profiles of 853 CRs were extracted from the normalized TCGA-BRCA matrix based on the obtained CR names. In the training set, DEGs related to CRs were performed by the “limma” package. Differential expression was set as the adjusted *p* value less than 0.05, and the absolute value of log2FC (fold change) was greater than 1. The result of the analysis was visualized in a volcano map utilizing the “ggplot2” *R* package.

### 2.3. Biology Function Enrichment

ClusterProfiler was conducted to analyze gene oncology and the KEGG pathway, and this procedure aims at discovering the potential mechanism of the CR-related DEGs. In addition, the enrichment analysis results were visualized via circos diagrams.

### 2.4. Signature Model Construction and Validation

Batch Cox regression was applied to screen the prognosis-related DEGs of CRs in the training set. And the important candidate genes were analyzed by the multivariate Cox analysis for identifying independent prognosis risk factors. Based on the multivariate regression results, we then developed an optimal signature to predict prognosis based on the coefficients (Coef). The model was constructed as the risk formula: risk score = A gene × Coef + B gene × Coef + … + X gene × Coef. The forest map was plotted by the “forestplot” *R* package. The area under curve of ROC will be used to evaluate the model in the training and validation set, respectively.

### 2.5. Signature Prognostic Value Evaluation

Other clinical variables also affect patient clinical prognosis, and when compared with risk score, if risk score is also associated with patients outcomes, here, we use Cox regression model to demonstrate it, and we also conduct subgroup analyses of risk score and survival status.

### 2.6. Immune Cell Infiltration and Immunotherapy Efficacy Estimation

To evaluate the association between this signature and tumor-infiltrating immune cells, CIBERSORT was used to analyze the proportion of various TICs between high-score and low-score patients in the TCGA training set. We also use the tumor immune dysfunction and exclusion (TIDE) indicator to estimate ICB therapy response in BC patients. Among them, lower TIDE scores mean that tumor cells have less chance with immune escape, indicating a higher response rate to ICBs therapy. Finally, we assessed the correlation between this signature and immunotherapeutic markers and ICB-related genes via Wilcoxon test.

### 2.7. Predictive Nomogram Establishment and Evaluation

A nomogram, a visual scoring system, was established in the training set by integrating the risk score of CR-related gene signature and clinicopathological variables to be used to evaluate the OS of the BC patients. At the same time, the bootstrap method was used to calculate the concordance index (C-index), ROC, and calibration curves. Besides, we further utilized the Kaplan–Meier analysis for OS, DSS, and PFS to prove the clinical prognostic value of the nomogram. All the analyses and plots were done by the R software and suitable *R* packages.

## 3. Results

### 3.1. CR-Related DEGs and Function Enrichment

**Figure 1:**
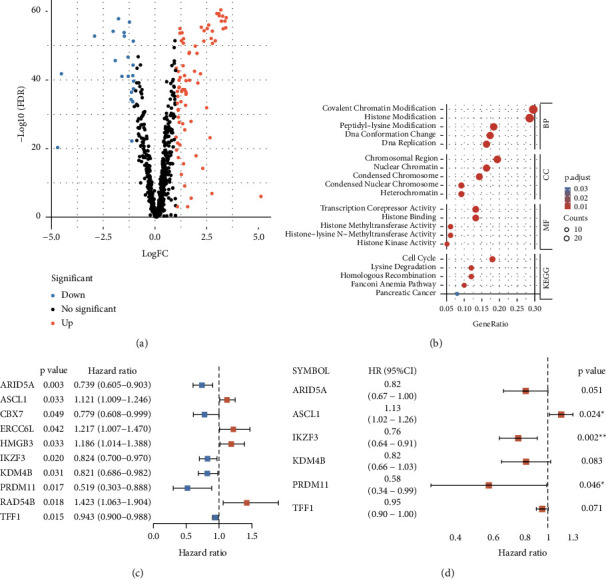
Differential expression analysis and the construction of CR-related prognosis signature in the TCGA training set. Volcano map of the CR-related DEGs. Red represents the up-regulated genes; blue represents the down-regulated genes (|logFC| > 1, FDR *q* value <0.05) (a). GO enrichment analysis (consisting of BP, CC and MF) and KEGG pathway enrichment analysis for the CR-related DEGs (*p* < 0.05, FDR *q* value <0.05) (b). Univariate Cox regression analysis selected 10 CR-related genes correlated with OS (c). Multivariate Cox regression analysis identified a 6-gene (ARID5A, ASCL1, IKZF3, KDM4B, PRDM11, and TFF1) prognostic signature (d). ^*∗*^*p* < 0.05 and ^*∗∗*^*p* < 0.01.

In the training dataset, a total of 98 CR-related DEGs (Figure 1(a)) (Supplementary Table 2) were screened from 1154 BC tissue samples and 113 normal tissue samples. We predicted the biological mechanisms of 98 CR-related DEGs. GO enrichment analysis showed that the most highly enriched molecular functions (MF) of these DEGs were histone binding, transcription corepressor activity, and histone kinase activity. Cellular components (CC) of these DEGs were covalent chromatin modification, histone modification, and DNA replication. The biological process (BP) of these DEGs was remarkably involved in covalent chromatin modification, histone modification, and DNA replication. Moreover, they are also enriched in the cell cycle, homologous recombination, and lysine degradation by KEGG enrichment analysis (Figure 1(b)).

### 3.2. Prognostic Signature Construction Based on the CR-Related Gene

We used 98 CR-related DEGs to build the prognostic signature in the TCGA training set. First, ten important CR-related genes associated with OS in BC patients were selected (Figure 1(c)). Then, six hub CR-related genes (ARID5A, ASCL1, IKZF3, KDM4B, PRDM11, and TFF1) were identified as associated with the OS and contributed to the risk signature (Figure 1(d)) (Table 1). The risk score = (−0.201073277) *∗*ARID5A + 0.1231206 *∗*ASCL1 +  (−0.268713644) *∗*IKZF3  +  (−0.197882736) *∗*KDM4B + (−0.538035757) *∗* PRDM11 + (−0.050431061) *∗* TFF1.

### 3.3. Evaluation and Validation of Signature Efficacy

**Figure 2:**
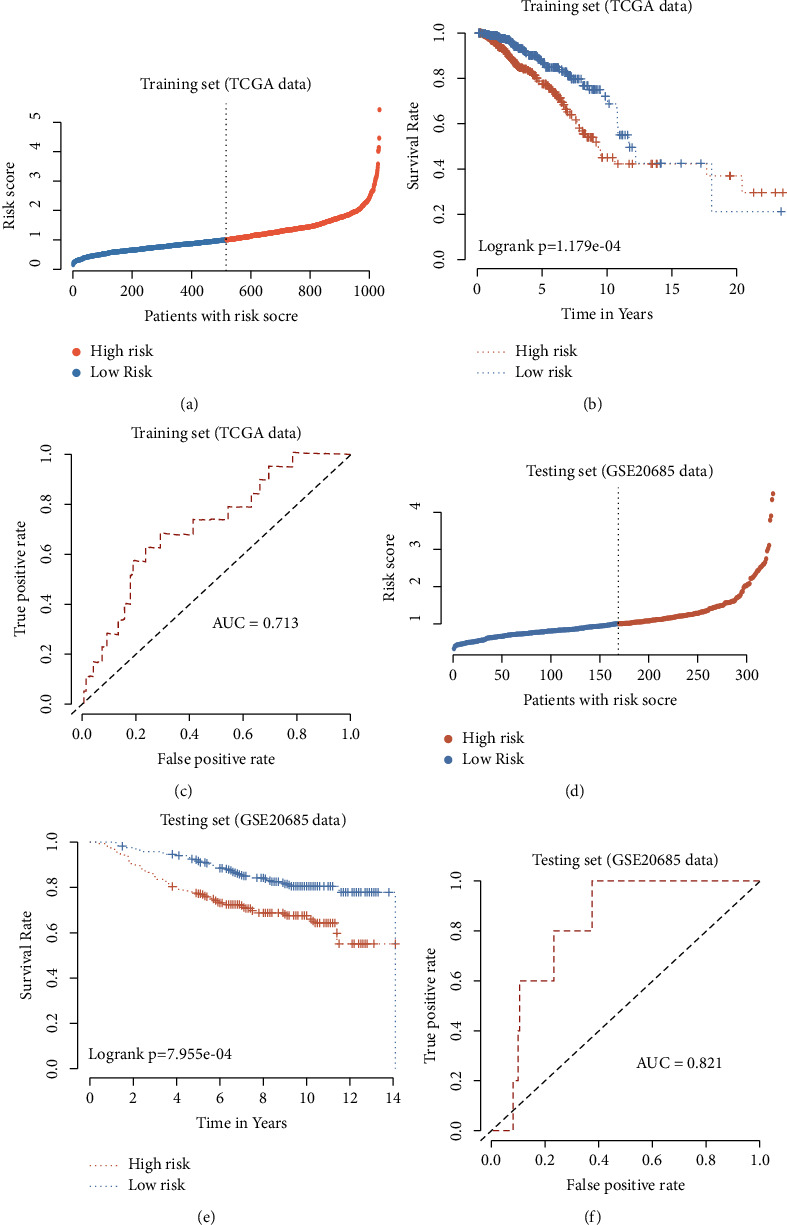
Validation of the CR-related prognostic signature in BC patients. Risk score distribution for patients in low- and high-risk groups from the TCGA training set (a). Kaplan–Meier survival analysis of OS between the low-and high-risk groups from the TCGA training set (b). AUC in ROC analysis for risk scores predicting the OS from the TCGA training set (c). Risk score distribution for BC patients in low- and high-risk groups from the GEO validation set (d). Kaplan–Meier survival analysis of OS between the low- and high-risk groups from the GEO validation set (e). AUC in ROC analysis for risk scores predicting the OS from the GEO validation set (f).

The high-risk group was defined as risk score more than the median value, others were defined as low-risk groups, respectively, in the training dataset. Then, the risk score distribution between the two groups was compared (Figure 2(a)). Patients with high risk have a poor outcome while the low-risk patients always suggest a better prognosis (*p*=1.179*e* − 04) (Figure 2(b)). Moreover, the area under the ROC curve (AUC) was 0.713, which proved the predictive efficacy of our signature for predicting the OS in the training set (Figure 2(c)), which was also validated in the GEO. The risk score distribution in the two groups is shown in Figure 2(d). In addition, patients with low risk also have a better prognosis (*p*=7.955*e* − 04) (Figure 2(e)), and the results further supported the above conclusion: AUC is equal to 0.821 (Figure 2(f)).

### 3.4. Applicability of the CR-Related Gene Signature

**Figure 3:**
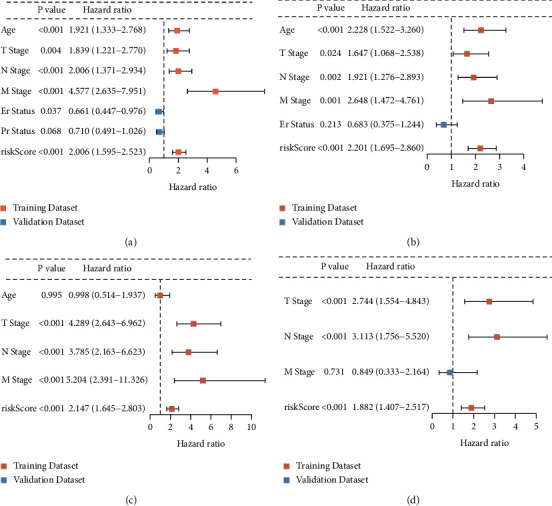
Independent prognostic analysis of the clinical traits and risk score in BC patients. Univariate and multivariate Cox regression analyses of the OS in the TCGA training set (a) and (b). Univariate and multivariate cox regression analyses of the OS in the GEO validation set (c) and (d).

We integrated the signature with other clinical risk factors in the total data set to further assess the independent prognostic value of the risk model for BC. As shown in the TCGA training dataset, age, TNM stage, ER status, and risk score were associated with the OS (*p* < 0.05), and risk score was an independent prognostic factor (*p* < 0.05) (Figures 3(a) and 3(b)). Additionally, in the GEO validation dataset, analysis results also support the abovementioned conclusions (Figures 3(c) and 3(d)).

### 3.5. Subgroup Analysis to Evaluate Gene Signature

**Figure 4:**
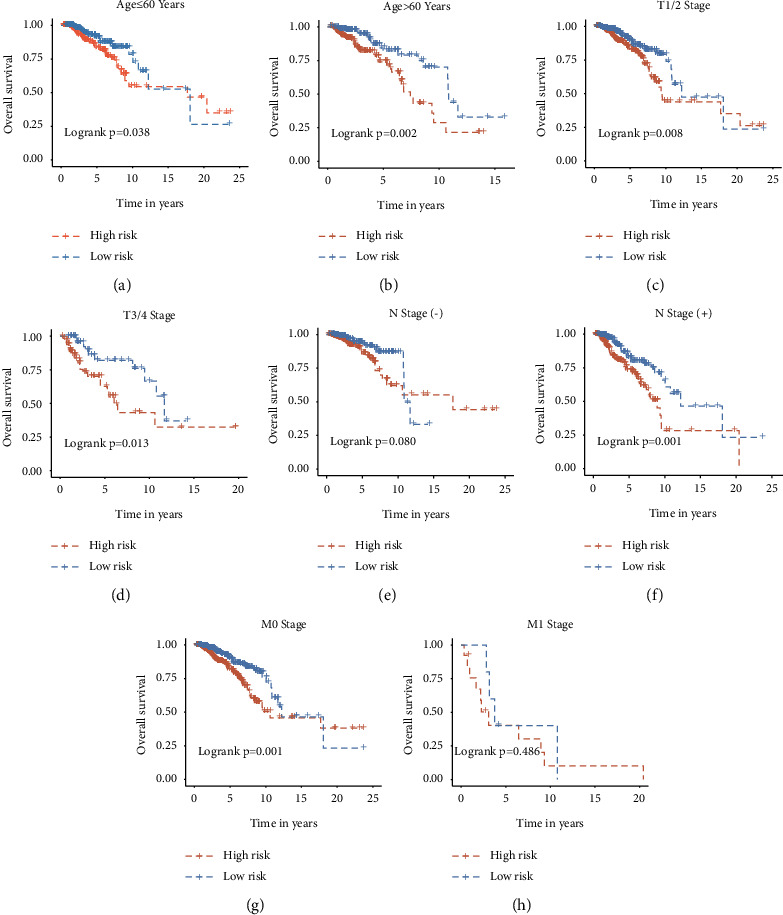
Kaplan–Meier survival subgroup analysis in BC patients from the TCGA training set based on the 6-gene signature stratified by clinical characteristics. The OS differences between low- and high-risk group stratified by age (≤60 years and >60 years, respectively) (a) and (b). The OS differences between low-and high-risk group stratified by T stage (T1-2 and T3-4, respectively) (c) and (d). The OS differences between low- and high-risk group stratified by N (−) and N (+) stage. (N0 and N1–3, respectively) (e) and (f). The OS differences between low- and high-risk group stratified by M stage (M0 and M1, respectively) (g) and (h).

We performed survival subgroup analysis in the TCGA training set to demonstrate that the signature is associated with clinical features. First of all, the clinical patients were classified into two groups, such as age ≤60 vs. >60 groups, the T1/2 stage vs. T3/4 stage, the N (−) stage vs. N (+) stage (N0 and N1–N3, respectively), and the M0 stage vs. M1 stage. Subgroup analysis suggests that subgroup patients also have survival rate differences in age, T stage, N1–N3 stage, and M0 stage between high- and low-risk groups. However, patients with the N0 or M1 stage has no significance (*p*=0.080 and *p*=0.486, respectively) (Figures 4(a)–4(h)).

### 3.6. Immune Cell Infiltration and Immunotherapy Efficacy Estimation for the Signature

**Figure 5:**
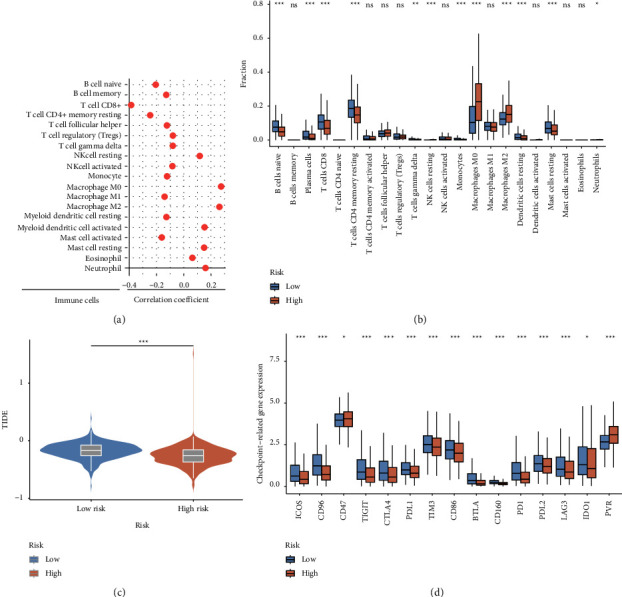
The estimation of immune cells infiltration and immunotherapy prediction for the prognostic model in the TCGA training set. Ns, not significant; ^*∗*^*p* < 0.05; ^*∗∗*^*p* < 0.01; ^*∗∗∗*^*p* < 0.001. The correlation analysis between the risk score and 19 tumor-infiltrating immune cells evaluated by CIBERSORT algorithm (*p* < 0.05) (a). The differences of 22 tumor-infiltrating immune cells between low- and high-risk groups evaluated by CIBERSORT algorithm (b). Violin plot showing the differential TIDE scores between the high-risk and low-risk groups (c). Box plot showing the expression of immune checkpoint-related markers in low-and high-risk groups (d).

As for the relationship between the signature and TME, 19 of the 22 TICs were significantly related to the risk score (Figure 5(a)). Here, the CD8^+^ T cells, resting memory CD4^+^ T cells, regulatory T cells (Tregs), gamma delta T cells, follicular helper T cells, M1 macrophages, memory B cells, naive B cells, activated NK cells, monocytes, resting myeloid dendritic cells, and activated mast cells were negatively correlated with the risk score, while the M0 macrophages, M2 macrophages, resting NK cells, activated myeloid dendritic cells, resting mast cells, eosinophils, and neutrophils were positively correlated with the risk score. Meanwhile, the proportion of 12 TICs did differ significantly between the two groups (*p* < 0.05, Figure 5(b)). The proportion of M0 and M2 macrophages was significantly higher in the high-risk group, while the proportion of plasma cells, naive B cells, CD8^+^ T cells, gamma delta T cells, resting memory CD4^+^ T cells, resting NK cells, and resting mast cells was significantly higher in the low-risk group.

Regarding the CR-related signature's potential for predicting response to immunotherapy in BC patients, the result showed that the low-risk group had a higher TIDE score (*p* < 0.001, Figure 5(c)), meaning a poor response to immunotherapy. Likewise, 13 immune checkpoint molecules (CTLA4, PDL1, PD1, PDL2, LAG3, TIM3, CD86, BTLA, ICOS, CD96, CD160, TIGIT, and IDO1) are also positive with low-risk group gene expression (*p* < 0.05, Figure 5(d)). Taken together, these results illustrated the importance of the risk score in breast cancer immunotherapy prediction.

### 3.7. Nomogram for Clinical Decision

**Figure 6:**
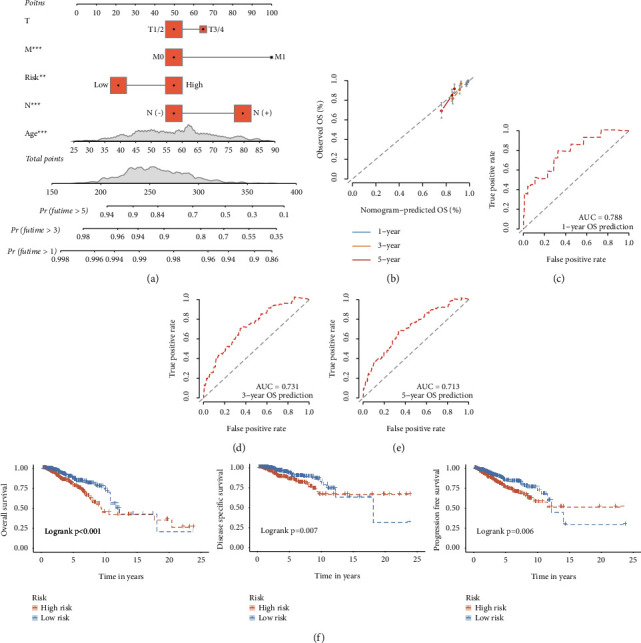
Construction and evaluation of a nomogram in the TCGA training set. The nomogram consists of age, T stage, N stage, M stage and risk score to predict the probability of 1-, 3-, and 5-year OS in BC patients (a). Calibration curves of 1-, 3- and 5- year OS in BC patients predicted by the Nomogram (b). ROC curves of 1-, 3-, and 5-year OS predicted by the nomogram (c)–(e). Kaplan–Meier survival curves for OS, DSS and PFS in BC patients based on the nomogram (f).

A visualized nomogram was built and was used to predict OS probability at three periods (1, 3, and 5 years) (Figure 6(a)). The concordance C-index was 0.734, which illustrated a good ability in predicting OS for BC patients. Furthermore, we found that the calibration curves indicated that the predicted curve is close to the ideal curve (Figure 6(b)). At the same time, the ROC curves of this nomogram also showed a good accuracy to individually predict OS for BC patients (AUC = 0.788, 0.731, and 0.713, respectively) (Figures 6(c)–6(e)). Lastly, we also assessed the prognostic value of the nomogram, finding that it was remarkably associated with OS, DSS, and PFS (*p* < 0.05) (Figure 6(f)).

## 4. Discussion

A significantly poor prognosis for the stage IV female BC patients who were diagnosed between 2007 and 2013 can be observed [24]. To improve the survival rate of BC patients, an increasing number of genetic signatures have been established. For example, Shi et al. [25] developed a five-mRNA signature model based on the ceRNA network for predicting the survival of BC. However, few studies have focused on prognostic signatures based on key genes in CRs that have been shown to have potential as prognostic markers. What's more, studies have shown that epigenetic factors and mechanisms can be involved in regulating the TME and the ICBs response. In lung adenocarcinomas, ASF1A deficiency could sensitize lung adenocarcinomas to anti-PD-1 therapy by inducing immunogenic M1-like macrophage differentiation and enhancing T cell activation of the TME [26]. Then, in checkpoint-blocked refractory mouse melanoma, histone demethylase LSD1 depletion enhanced tumor immunogenicity and T-cell infiltration in poorly immunogenic tumors and elicited a significant response to anti-PD-1 treatment [27]. Thus, exploring and evaluating the CR-related gene expression in BC patients is important. In our study, we constructed a CR-related gene signature, a useful tool to predict patients' outcomes and immunotherapy sensitivity.

The CR-related gene signature has 6 hub genes; ASCL1, as one of the 6 genes, was positively associated with outcome, while the levels of ARID5A, IKZF3, KDM4B, PRDM11, and TFF1 were negatively associated with survival. As the results demonstrated, the model could predict the patient's outcome.

The ROC curves also confirmed the favorable predictive performance of this signature. Besides, the independent prognostic analysis determined that this signature, age, and TNM stage were independent predictors for BC prognosis. As expected, survival subgroup analysis also suggests the effective prediction ability of the signature in subgroups. Next, we found that patients with high-risk conditions will obtain a better response to immunotherapy and were more suitable for ICB therapy. Moreover, a nomogram model consisting of clinical factors and signature was established, which showed good power and accuracy with a high AUC value and C-index in BC patients. Therefore, the CR-related gene signature was a reliable model to predict prognosis and immunotherapy efficacy, which might have potential implications in clinical practice for BC.

All six genes involved in the signature model are associated with chromatin regulation or tumorigenesis. Achaete-scute complex homolog 1 (ASCL1) is a key regulator of neuroendocrine differentiation [28]. ASCL1 was highly expressed in classic small cell lung cancer (SCLC); additionally, it was a key driver of tumorigenesis in classic SCLC and correlated with the survival and development of lung cancers with neuroendocrine (NE) features [29]. Jiang et al. [30] confirmed that when ASCL1 was successfully overexpressed with SV40 large T-antigen, it could synergistically inhibit retinoblastoma protein and p53 to promote the development of aggressive adenocarcinoma with NE characteristics; however, in the developing mouse lung, knockout of ASCL1 resulted in specific ablation of lung NE cells. Moreover, in recent studies, ASCL1 demonstrated that it is involved in the differentiation, cell proliferation, and E-cadherin expression of NE cells in SCLC cell lines by regulating the Wnt signaling pathway [31]. Our work also found that ASCL1 was a risk factor for BC prognosis patients, implying that it might promote breast cancer tumorigenesis. ARID5A (AT-rich interactive domain-containing protein 5a) is one of the Arid family of proteins and possesses the ability to bind nucleic acids, which exist in the nucleus under normal conditions [32]. Meanwhile, ARID5A has been shown to mainly regulate inflammatory and autoimmune disease development by regulating the expression of Interleukin-6 (IL-6) mRNA [33]. Subsequent research revealed that ARID5A could regulate IL-6 mRNA stability through NF-*κ*B and MAPK signaling pathways [34]. As for IKZF3 (Aiolos), it belongs to the family of Cys2-His2 zinc finger proteins, which is a lymphocyte lineage transcription factor necessary for the survival of the malignant [35]. For multiple myeloma, immunomodulatory drugs, such as thalidomide could activate E3-ubiquitin ligases and induce degradation of key transcription factors to exert direct antimyeloma effects and promote the survival of myeloma [36]. Then, IKZF3 was a frequently mutated tumor suppressor gene in acute lymphoblastic leukemia (ALL), and its deletion could block lymphocytic lineage differentiation and increase the susceptibility to developing ALL [37]. Consistently, we also found that IKZF3 was a protective factor for BC and facilitated the prognosis of BC patients. Lysine-specific histone demethylase 4B (KDM4B) is a histone demethylase for H3K9me3 [38]. According to the genome-wide analysis, KDM4B might be a cancer-specific regulator of alternative splicing by regulating additional alternative splicing-related genes involved in tumorigenesis [39]. In breast cancer, studies have revealed that KDM4B not only antagonizes H3K9 tri-methylation in peripheral heterochromatin and affects H3K4/H3K9 methylation but also plays a role in estrogen receptor *α*-regulated breast cancer development and mammary epithelial cells proliferation [40, 41]. At the same time, KDM4B was the first identified androgen receptor AR)-regulated demethylase with effects on AR signaling and turnover and might be a therapeutic target for prostate cancer [42]. The PR-domain (PRDM) family of genes and the putative transcriptional regulatorsbelong to the SET domain family of histone methyltransferases, which can directly catalyze histone lysine methylation or work by recruiting transcriptional cofactors [43]. Some of the PRDMs are deregulated in cancer and act as tumor suppressors or oncogenes, especially in hematologic malignancies and solid cancers [44]. In diffuse large B-cell lymphomas (DLBCLs), the study showed that the overexpression of PRDM11 (PR-domain family member Prdm11) could induce apoptosis in the E*μ*-Myc mouse model, then, the DLBCLs patients with low levels of PRDM11 correlate with shorter overall survival [45]. Additionally, PRDM11 was also identified as a novel locus associated with forced vital capacity, which could be a new target for lung diseases in a genome-wide association analysis [46]. TFF1 (trefoil factor 1), one of the trefoil factor family (TFF), is a small molecule peptide and prevalent in the mucosal environment [47]. In human gastric cancer, it is widely accepted that TFF1 is markedly low-expressed and functions as a gastric tumor suppressor [48]. In BC, although the serum and tissue levels of TFF1 are typically overexpressed [49], many clinical studies have also reported that TFF1 deficiency increases tumorigenicity of human breast cancer cells, and TFF1 expression in BC has an effect on good clinical outcomes for patients [50, 51]. Taken together, all 6 CR-related genes have been reported as taking part in the development of tumors, playing a role in cancer suppression or carcinogenesis. However, the prognostic role in tumors is still less elucidated, and more studies and investigations are needed to understand their prognostic value and mechanisms.

To our knowledge, this is the first time to establish and validate the chromatin regulator-related gene prognostic signature using a large sample size and a high AUC value for breast cancer. Regardless, several limitations can be further improved in the study. In the first place, although our results showed the predictive potentiality and clinical value of our signature, the potential mechanisms of these 6 CR-related genes in BC still require more in-depth experimental investigation. Secondly, the data and information from a total of 1,362 BC patients in the public database used to build the prognostic signature and validate the predictive efficiency of this model are inadequate; therefore, prospective clinical studies are supposed to further confirm our findings.

In conclusion, we identified CR-related DEGs and their predictive ability of prognosis in breast cancer. After that, a novel 6-gene signature model using CR-related DEGs was developed and validated to predict the OS and immunotherapeutic sensitivity for BC patients. Furthermore, a nomogram integrating this novel gene signature and clinical factors was constructed to accurately predict the prognosis for breast cancer, which might provide individualized treatment and aid clinical decision-making for BC patients through prospective validation experiments in the future.

## Figures and Tables

**Table 1:** Six CR-related prognostic genes significantly associated with OS in breast cancer patients.

Gene	Multivariate cox regression analysis				
	Coef	HR	HR 0.95L	HR 0.95 H	*p* value
ARID5A	−0.201073277	0.8178525	0.668022015	1.001288425	0.051475659
ASCL1	0.1231206	1.131020814	1.016273871	1.258723773	0.02408809
IKZF3	−0.268713644	0.764362104	0.642458524	0.909396333	0.002434433
KDM4B	−0.197882736	0.820466059	0.655829072	1.026432927	0.083334851
PRDM11	−0.538035757	0.583894036	0.344413023	0.989893597	0.045751465
TFF1	−0.050431061	0.950819475	0.900181977	1.004305459	0.070902358

CRs, chromatin regulators; OS, overall survival; Coef, *β* coefficient; HR, hazard ratio.

## Data Availability

The data used in this study can be obtained from the public databases, namely, The Cancer Genome Atlas (TCGA) and Gene Expression Omnibus (GEO).

## Supplementary Materials

Supplementary Materials Supplementary Table 1: clinical information of BC patients in this study. Supplementary Table 2: chromatin regulators-related differentially expressed genes in breast cancer.
