# Supplementary material for: Chromatin Regulator-Related Gene Signature for Predicting Prognosis and Immunotherapy Efficacy in Breast Cancer
Source: J Oncol. 2023 Jan 30;2023:2736932. doi: 10.1155/2023/2736932 (PMC9902130; doi:10.1155/2023/2736932)
Supplement: Supplementary Materials — Supplementary Table 1: clinical information of BC patients in this study. Supplementary Table 2: chromatin regulators-related differentially expressed genes in breast cancer. [file 2736932.f1.zip › Supplementary Table1.docx]

**Supplementary Table 1 | Clinical information of BC patients in the this study.**

| **Variables** | **TCGA-BRCA**  **(Training set)** | **GEO-GSE20685**  **(Validation set)**  **GSE14520**  **(Validation)** |
| --- | --- | --- |
| **Total** | 1035 | 327 |
| **Age** |  |  |
| ≤60 | 579 | 282 |
| >60 | 456 | 45 |
| **T stage** |  |  |
| T1 | 276 | 101  - |
| T2 | 591 | 188  - |
| T3 | 129 | 26 |
| T4 | 63 | 12  - |
| Unknown | 3 | -  - |
| **N stage** |  |  |
| N0 | 484 | 137  - |
| N1 | 352 | 87  - |
| N2 | 110 | 63 |
| N3 | 72 | 40  93 |
| Unknown | 17 | -  77 |
| **M stage** |  | 49 |
| M0 | 852 | 319  0 |
| M1 | 21 | 8 |
| Unknown | 162 | -  - |
| **ER** |  | - |
| Positive | 770 | -  - |
| Negative | 222 | -  - |
| Unknown | 43 | -  - |
| **PR** |  |  |
| Positive | 673 | - |
| Negative | 317 | -  121 |
| Unknown | 45 | -  100 |
| **OS event** |  |  |
| Alive | 889 | 282  - |
| Dead | 146 | 45  - |
| **DSS event** |  |  |
| Alive | 935 | - |
| Dead | 81 | - |
| Unknown | 19 | - |
| **PFS event** |  |  |
| Alive | 892 | - |
| Dead | 143 | - |

**Abbreviations:** BC, breast cancer; TCGA, the cancer genome atlas; GEO, gene expression omnibus; T, tumor depth; N , lymph node metastasis; M,distant metastasis; ER,estrogen receptor; PR , progesterone receptor; OS, overall survival; DSS, disease-specifific survival; PFS,progression-free survival .
