# Supplementary material for: Chromatin Regulator-Related Gene Signature for Predicting Prognosis and Immunotherapy Efficacy in Breast Cancer
Source: J Oncol. 2023 Jan 30;2023:2736932. doi: 10.1155/2023/2736932 (PMC9902130; doi:10.1155/2023/2736932)
Supplement: Supplementary Materials — Supplementary Table 1: clinical information of BC patients in this study. Supplementary Table 2: chromatin regulators-related differentially expressed genes in breast cancer. [file 2736932.f1.zip › Supplementary Table2.docx]

**Supplementary Table 2 | Chromatin regulators-related differentially expressed genes in breast cancer.**

| **Gene** | **conMean** | **treatMean** | **logFC** | **p-Value** | **FDR q-Value** |
| --- | --- | --- | --- | --- | --- |
| APOBEC2 | 4.304268071 | 0.165070788 | -4.704611195 | 1.41E-21 | 4.94E-21 |
| APOBEC3A | 0.213007276 | 0.635875249 | 1.577841042 | 0.000693309 | 0.000871271 |
| APOBEC3B | 1.095570311 | 6.169760641 | 2.493532445 | 1.91E-33 | 1.24E-32 |
| ARID5A | 25.47023789 | 11.70361252 | -1.121858523 | 1.65E-23 | 6.32E-23 |
| ARID5B | 26.95147608 | 9.650846141 | -1.481636947 | 5.57E-55 | 1.64E-53 |
| ASCL1 | 0.076213104 | 2.645605777 | 5.117415217 | 5.90E-07 | 8.98E-07 |
| ASCL2 | 0.682818902 | 2.700426427 | 1.983612342 | 4.00E-19 | 1.24E-18 |
| ASF1B | 1.584864239 | 12.59713598 | 2.9906646 | 1.24E-62 | 4.37E-60 |
| ATAD2 | 3.541841811 | 13.29885703 | 1.908730576 | 2.10E-44 | 2.90E-43 |
| AURKA | 1.117367913 | 9.4587915 | 3.081551573 | 2.08E-62 | 4.88E-60 |
| AURKB | 0.665564303 | 6.521898516 | 3.292642029 | 2.75E-57 | 1.21E-55 |
| BARD1 | 1.50672668 | 3.283712875 | 1.123910249 | 6.53E-33 | 4.15E-32 |
| BRCA1 | 1.194594242 | 2.548381466 | 1.093060577 | 6.62E-23 | 2.44E-22 |
| BRCA2 | 0.379134596 | 1.054570541 | 1.475873587 | 3.62E-34 | 2.43E-33 |
| BUB1 | 0.526452689 | 4.85926405 | 3.20636204 | 8.51E-60 | 6.94E-58 |
| CBX2 | 0.760198493 | 4.805547046 | 2.660252601 | 1.70E-24 | 6.91E-24 |
| CBX4 | 6.683101479 | 21.15813303 | 1.662622645 | 4.63E-50 | 9.07E-49 |
| CBX7 | 11.15829191 | 3.96179915 | -1.493888552 | 4.07E-56 | 1.44E-54 |
| CBX8 | 1.185373453 | 3.186658504 | 1.426702769 | 4.01E-43 | 5.14E-42 |
| CDC6 | 0.8398086 | 6.323207052 | 2.912523994 | 1.52E-53 | 3.68E-52 |
| CDK1 | 1.467297365 | 12.61461687 | 3.103863205 | 1.09E-61 | 1.92E-59 |
| CDK5 | 2.77221708 | 7.196704357 | 1.37629616 | 3.26E-54 | 8.83E-53 |
| CHAF1A | 3.055992419 | 6.464412684 | 1.080878337 | 2.54E-38 | 2.18E-37 |
| CHAF1B | 1.823657179 | 4.625048038 | 1.342633801 | 6.52E-39 | 5.97E-38 |
| CHEK1 | 1.02284317 | 2.888994459 | 1.49798248 | 1.40E-41 | 1.57E-40 |
| CIT | 0.499696703 | 1.925547123 | 1.946143824 | 6.46E-52 | 1.34E-50 |
| DBF4 | 1.612599954 | 3.632243817 | 1.171472462 | 1.26E-39 | 1.20E-38 |
| DNAJC1 | 13.73913928 | 39.06949738 | 1.507751069 | 9.48E-38 | 7.60E-37 |
| DNMT3B | 0.807482156 | 2.189849165 | 1.439329218 | 7.31E-27 | 3.35E-26 |
| DUSP1 | 303.3423743 | 80.13181038 | -1.920500068 | 1.29E-47 | 2.21E-46 |
| ERCC6L | 0.255923778 | 1.746798038 | 2.770926718 | 1.47E-56 | 5.77E-55 |
| EXOSC4 | 7.183392407 | 19.44359325 | 1.436557626 | 7.06E-39 | 6.38E-38 |
| EZH1 | 13.15353255 | 5.592178621 | -1.233967958 | 1.89E-59 | 1.33E-57 |
| EZH2 | 1.340859148 | 6.312203365 | 2.234985991 | 7.23E-58 | 4.25E-56 |
| FBXL19 | 3.271442935 | 7.609518123 | 1.217877991 | 1.49E-42 | 1.78E-41 |
| FOXA1 | 23.89180617 | 79.82229478 | 1.740275832 | 2.24E-29 | 1.18E-28 |
| FOXO1 | 17.45375464 | 5.138557344 | -1.764102138 | 1.37E-60 | 1.38E-58 |
| FOXP3 | 0.592774306 | 2.486713197 | 2.068685304 | 7.57E-41 | 7.73E-40 |
| GADD45G | 4.18984449 | 10.90756408 | 1.380360346 | 9.64E-17 | 2.64E-16 |
| GLYATL1 | 0.456719858 | 1.533606091 | 1.747546548 | 1.90E-06 | 2.79E-06 |
| HDAC11 | 4.663596956 | 9.60391365 | 1.042179321 | 7.22E-21 | 2.50E-20 |
| HDGF | 52.37172938 | 120.8646301 | 1.206531965 | 2.02E-57 | 1.01E-55 |
| HELLS | 0.618304435 | 1.957676563 | 1.662753172 | 1.03E-41 | 1.17E-40 |
| HJURP | 0.399475853 | 4.339123315 | 3.441223381 | 5.81E-61 | 6.83E-59 |
| HMGA1 | 22.63023428 | 63.7077048 | 1.493216342 | 1.86E-37 | 1.46E-36 |
| HMGB2 | 22.41344714 | 49.05923895 | 1.130160304 | 1.28E-30 | 7.10E-30 |
| HMGB3 | 4.210773841 | 21.58417235 | 2.357816477 | 2.11E-53 | 4.79E-52 |
| IDH2 | 34.93087982 | 85.28995276 | 1.28787282 | 2.60E-38 | 2.21E-37 |
| IKZF3 | 1.061930176 | 2.24849535 | 1.082270991 | 0.000672375 | 0.000846472 |
| JADE1 | 8.548281381 | 4.130269096 | -1.049398618 | 2.02E-53 | 4.74E-52 |
| KAT2B | 7.743431434 | 3.13823138 | -1.303021301 | 1.19E-48 | 2.11E-47 |
| KDM4B | 4.987663274 | 10.91619297 | 1.130033829 | 1.38E-18 | 4.13E-18 |
| KMT5C | 1.52965893 | 3.090830281 | 1.014784428 | 1.16E-24 | 4.74E-24 |
| LMNB1 | 3.553233914 | 18.39765943 | 2.372317665 | 1.04E-56 | 4.29E-55 |
| LMNB2 | 5.698968416 | 14.60718038 | 1.357905019 | 4.73E-46 | 7.09E-45 |
| LRWD1 | 1.636150612 | 3.561621131 | 1.122228499 | 2.38E-44 | 3.23E-43 |
| MAZ | 9.398654248 | 22.743557 | 1.2749318 | 1.49E-53 | 3.68E-52 |
| MECOM | 3.216048478 | 1.445693935 | -1.153526999 | 6.96E-36 | 5.01E-35 |
| MOCS1 | 12.17092712 | 2.994775742 | -2.022919191 | 1.71E-56 | 6.33E-55 |
| MRGBP | 4.351018885 | 8.847644791 | 1.023940188 | 2.55E-45 | 3.74E-44 |
| NAP1L2 | 3.189298369 | 1.052906313 | -1.598862001 | 7.17E-43 | 8.71E-42 |
| NCOA7 | 14.49964307 | 6.887519 | -1.073961088 | 3.08E-35 | 2.17E-34 |
| ORC1 | 0.505373114 | 2.317527201 | 2.197165453 | 1.19E-43 | 1.56E-42 |
| PAGR1 | 2.867681248 | 6.843755189 | 1.254903481 | 2.92E-35 | 2.08E-34 |
| PARP1 | 18.67828558 | 43.03269748 | 1.204071237 | 7.12E-56 | 2.28E-54 |
| PBK | 0.698351114 | 7.26611046 | 3.379158822 | 8.87E-60 | 6.94E-58 |
| PCGF2 | 8.82420608 | 19.1804412 | 1.120097518 | 1.00E-32 | 6.32E-32 |
| PCNA | 33.68110177 | 87.03609705 | 1.369674531 | 8.40E-53 | 1.85E-51 |
| PIWIL4 | 1.158970471 | 0.527799103 | -1.134783005 | 4.40E-38 | 3.61E-37 |
| PPP4C | 19.40790437 | 48.03711221 | 1.307505078 | 4.97E-56 | 1.67E-54 |
| PRC1 | 1.801231375 | 10.8750939 | 2.59397244 | 1.47E-58 | 9.45E-57 |
| PRDM11 | 1.614861765 | 0.774133634 | -1.060756137 | 3.01E-46 | 4.71E-45 |
| PRKCA | 2.017683517 | 0.959550001 | -1.072270008 | 4.16E-43 | 5.23E-42 |
| RAC3 | 5.70190354 | 14.42341081 | 1.338896831 | 1.14E-28 | 5.79E-28 |
| RAD51 | 0.605841022 | 3.480069274 | 2.522104852 | 2.20E-57 | 1.04E-55 |
| RAD54B | 0.315482892 | 0.883089678 | 1.48499818 | 3.21E-38 | 2.69E-37 |
| RAD54L | 0.381555383 | 2.502361328 | 2.713325737 | 2.76E-54 | 7.78E-53 |
| RARA | 11.93296327 | 27.47646416 | 1.203244015 | 2.20E-22 | 8.04E-22 |
| RCC1 | 7.297950735 | 15.63231905 | 1.0989685 | 7.73E-37 | 5.80E-36 |
| RCOR2 | 0.497909675 | 2.46367761 | 2.306857527 | 2.31E-15 | 5.87E-15 |
| RMI1 | 2.870453381 | 6.422221902 | 1.161793889 | 9.65E-42 | 1.12E-40 |
| RPS6KA3 | 9.824589292 | 4.779381169 | -1.039573267 | 2.08E-41 | 2.25E-40 |
| SAP30 | 4.722696717 | 9.95680408 | 1.076071851 | 8.67E-35 | 5.99E-34 |
| SATB1 | 3.947488345 | 1.915744696 | -1.043029695 | 4.01E-39 | 3.72E-38 |
| SMYD1 | 5.923731522 | 0.25779317 | -4.522220309 | 1.19E-43 | 1.56E-42 |
| SMYD3 | 0.734627143 | 2.29453833 | 1.643119799 | 9.58E-50 | 1.78E-48 |
| SP140 | 0.392655193 | 0.94915526 | 1.273381123 | 1.53E-13 | 3.47E-13 |
| TDRKH | 2.657931381 | 5.922235363 | 1.155837968 | 2.00E-39 | 1.88E-38 |
| TFF1 | 59.36440165 | 399.0977154 | 2.749072049 | 7.61E-08 | 1.24E-07 |
| TFPT | 7.382969781 | 16.571315 | 1.166414932 | 4.40E-29 | 2.27E-28 |
| TLE4 | 4.185290592 | 1.706607418 | -1.294196571 | 6.66E-43 | 8.24E-42 |
| TONSL | 0.910302543 | 3.713790842 | 2.028474549 | 9.00E-50 | 1.71E-48 |
| TOP2A | 2.600795545 | 27.81007099 | 3.418582533 | 1.13E-57 | 6.12E-56 |
| TOX3 | 2.191878918 | 5.91661135 | 1.432603027 | 4.24E-10 | 7.66E-10 |
| TTK | 0.559501229 | 3.830296979 | 2.775243049 | 9.93E-53 | 2.12E-51 |
| UBE2T | 2.592213842 | 23.49453375 | 3.180068495 | 5.28E-64 | 3.72E-61 |
| UHRF1 | 0.477826829 | 4.604824723 | 3.268586477 | 1.69E-61 | 2.38E-59 |
| ZBTB16 | 4.72054105 | 0.623440462 | -2.920628531 | 5.14E-55 | 1.58E-53 |
